# Characterization of Intelligence in Children with Exotropia

**DOI:** 10.3390/ijerph16173008

**Published:** 2019-08-21

**Authors:** Tao Sun, Zhonghao Wang, Tao Shen, Jianhua Yan, Chuanbo Xie, Xiuhong Li

**Affiliations:** 1 Department of Maternal and Child Health, School of Public Health, Sun Yat-sen University, Guangzhou 510080, Guangdong, China; 2 State key laboratory of Ophthalmology, Zhongshan Ophthalmic Center, Sun Yat-sen University, Guangzhou 510080, Guangdong, China; 3 State Key Laboratory of Oncology in South China, Collaborative Innovation Center for Cancer Medicine, Sun Yat-sen University Cancer Center, 651 East Dongfeng Road, Guangzhou 510080, Guangdong, China

**Keywords:** exotropia, intelligence, Wechsler Intelligence Scale for Children, perceptual reasoning, processing speed

## Abstract

The effect of exotropia on the intelligence of children is unknown. This study aimed to assess the intelligence in children with exotropia and investigate the influence of the main clinical indexes of strabismus on intelligence. Eighty-four participants aged 8–12 years were enrolled, including 37 patients with exotropia (exotropia group) and 47 normal individuals (normal group). Intelligence was assessed by the Wechsler Intelligence Scale for Children—Fourth Edition (WISC-IV), including the Verbal Comprehension Index (VCI), Perceptual Reasoning Index (PRI), Working Memory Index (WMI), Processing Speed Index (PSI), and Full-Scale Intelligence Quotient (FSIQ). The exotropia group had a significantly lower PRI score but a higher PSI score than the normal group. However, there was no significant difference in the WMI, VCI, and FSIQ between groups. Multiple linear regression showed that PRI–WMI and PRI–PSI differences were significantly lower in the exotropia group. Inter-subscale correlations analysis showed that the pattern of intelligence structure was different between groups. The type of exotropia, angle of deviation, duration of symptoms, and stereoacuity had no effect on the intelligence of children with exotropia. Children with exotropia had a relatively worse performance in the perceptual reasoning skill but a better processing speed and a different pattern of intelligence structure.

## 1. Introduction

Exotropia is a form of strabismus, characterized by an outward deviation of the eyes. Intermittent exotropia (X(T)) and constant exotropia (XT) are the two major types of exotropia [1]. Epidemiological studies show that the prevalence of exotropia is relatively higher in Asian children than in Western children [2,3,4,5,6,7,8]. In China, the prevalence of exotropia is reported as ranging from 0.16% to 4.57% in children [9,10]. Currently, corrective surgery is the main treatment for exotropia.

Visual perception affects oculo-motor, coordination ability, spatial working memory, and nonverbal matrix reasoning, which is closely related to children’s reading ability and mathematical ability [11]. Previous reports show that strabismus children cannot maintain long-term attention due to being prone to asthenopia, and their attention span is flawed [12,13]. Strabismus patients have a higher incidence of attention deficit hyperactivity disorder (ADHD) traits than normal individuals [14,15,16]. Due to deficits in depth perception and attention, children with strabismus may have a different intellectual performance than normal children. In a study of the Iranian population, Bagheri et al. have reported that patients with congenital strabismus (mean age = 18.4 ± 10.5 years, range: 4–63) had a lower mean intelligence quotient (IQ) score than the normal population [17]. By contrast, Ghaderpanah et al. adopted WPPSI (the preschool and primary scale of intelligence versions of Wechsler) test to evaluate 3 to 7-year-old strabismus Iranian children, and found that there was no difference in the verbal intelligence quotient, operational intelligence quotient, and total intelligence quotient between strabismus children and normal children, and no negative effect of strabismus on preschool children’s intelligence quotient was found [18]. Thus, the effect of strabismus on intelligence remains controversial.

In addition to appearance problems, exotropia can cause impairment of stereoscopic vision [19,20]. It has been shown that children without stereoscopic vision have a poor performance in the areas of visual-motor integration, constructive praxia, and non-verbal reasoning [21]. Compared to normal children, exotropia children have poor non-verbal performance, including constructive praxia, visual memory, and strategy formation [21]. Children with exotropia have cognitive deficits such as spatial perception, working memory, and strategy formation, which may lead to mental retardation and an abnormal intelligence structure. Based on the above observation, we hypothesized that exotropia may have an impact on the intelligence of exotropia children. However, there is no study focusing on the intelligence of exotropia children thus far. Both Bagheri and Mahboubeh found that exotropia patients have higher IQ scores than patients with other types of strabismus [17,18]. Nevertheless, they did not compare the difference between patients with exotropia and the normal population. Although this result implies that exotropia may affect the intelligence of exotropia children, the effect of exotropia on intelligence remains to be investigated. Therefore, this study aimed to characterize the intelligence of children with exotropia by the Wechsler Intelligence Scale for Children (WISC), one of the most widely used intelligence scales in clinical practice and research [22].

## 2. Methods

### 2.1. Participants

This was a cross-sectional, case-controlled study. A total of 37 child patients with exotropia treated at the Department of Strabismus and Amblyopia at the Zhongshan Ophthalmic Center (Guangzhou, China) were enrolled in this study. The inclusion criteria for the exotropia group were: 1) aged between 8 and 12 years; 2) diagnosis according to the Chinese expert consensus on strabismus classification (2015) [23]; 3) met surgical indications and had been scheduled for strabismus surgery; 4) with a best-corrected visual acuity of 20/20 in both eyes; 5) with a good general health condition; and 6) be able to undergo the intelligence test. Patients with other ophthalmic diseases, such as strabismus, amblyopia, glaucoma, a history of ophthalmic surgery, or other serious acute or chronic diseases, as well as mental illness, were excluded.

Meanwhile, 47 normal children were recruited from the internet as the control group. These children were examined by an ophthalmologist to exclude any visual deficit before enrollment. The inclusion criteria for the control group were: 1) aged between 8 and 12 years; 2) with the best-corrected visual acuity of 20/20 in both eyes; and 3) be able to undergo the intelligence test. Children with other ophthalmic diseases, such as strabismus, amblyopia, glaucoma, a history of ophthalmic surgery, or other serious acute or chronic diseases, as well as mental illness, were excluded.

This study was approved by the institutional review board of the Zhongshan Ophthalmic Center of Sun Yat-sen University (No.2018KYPJ062). Written informed consent was obtained from the patient.

### 2.2. Instruments

The demographic characteristics were collected from each participant using a self-constructed questionnaire, including age, gender, place of residence, education level of the mother, education level of the father, total monthly household income, with or without sibling(s), gestational age, delivery method, and admitted to NICU at birth.

The Wechsler Intelligence Scale for Children (WISC)-IV Chinese Version [24] was used to assess the intelligence of all participants by certified researchers. The scale measures the intellectual structure of 6–16-year-old children and adolescents. It adopts a deviation intelligence quotient, with a mean of 100 and a standard deviation of 15. WISC-IV includes 10 core tests and 4 supplementary tests. The scores of these subtests can be converted into four indexes: Verbal Comprehension Index (VCI), Perceptual Reasoning Index (PRI), Working Memory Index (WMI), and Processing Speed Index (PSI). Together, the four indexes provide the Full-Scale Intelligence Quotient (FSIQ). A subtest-level discrepancy comparison was calculated by the differences between each index (VCI−PRI, VCI−WMI, VCI−PSI, PRI−WMI, PRI−PSI, and WMI−PSI). The reliability of the WISC-IV Chinese Version in the subtests and FSIQ is 0.82–0.94 and 0.90–0.98, respectively, and the validity in the subtests and FSIQ is 0.68–0.86 and 0.78–0.91, respectively [25].

### 2.3. Procedures

Parents and children were informed about the purpose of the study and the process of evaluation. Researchers emphasized that participation was entirely voluntary and whether to participate or not had no impact on their surgery or treatment. Normal individuals were recruited in the same city by internet enrollment. The history of ophthalmopathy was collected by a medical history questionnaire filled in by their parents and the optometry report. Participants completed the intelligence test in a standard assessment room. All data were collected before the strabismus surgery.

### 2.4. Optometry Examinations

The data about duration of symptoms (from symptom onset to the intelligence test) and the history of ophthalmic surgery was collected by an inquiry from the parents of participants by two strabismus specialists. All the optometry examinations were performed in current refractive correction by at least two strabismus specialists. The angle of deviation was determined by the alternate prism cover test (APCT). Near stereoacuity was assessed using the Fly Stereo Acuity Test (Vision Assessment Co, Elgin, IL, USA) with the threshold of 400, 200, 100, and 60 arcsec, requiring 2 of the 2 presentations at each level to pass the level. If the child could not pass the 400 arcsec level, the near stereoacuity would be recorded as “none”. Distance stereoacuity was assessed using the Distance Randot Test (Stereo Optical Co, Chicago, IL, USA) with the threshold of 400, 200, 100, and 60 arcsec (requiring 2 of the 2 presentations at each level to pass the level) at a distance of 3 m. If the child could not pass the 400 arcsec level, their stereoacuity was recorded as “none” at the corresponding distance.

### 2.5. Statistical Analysis

The data of demographic and clinical variables were presented as the mean (SD) or number (percent) and were compared with a Student’s t-test or Chi-square test (Fisher’s exact test for any expected value lower than 5 was observed), respectively. On the basis of evaluating the distribution of data with the Kolmogorov–Smirnov test, simple and multiple linear regression models were used to test the differences between children with exotropia and healthy controls while significant covariates were adjusted. The same linear regression procedures were done to assess the differences in different types of exotropia, stereovision, strabismus angle, and duration of symptoms. Pearson correlation coefficient analyses were used to observe the correlated structure among WISC-IV domains, total score (FSIQ), and differences in intelligence structures. A *p*-value lower than 0.05 would be recognized as reaching significance in each test. All analyses were performed using IBM SPSS Version 20 (SPSS Statistics V20, IBM Corporation, Somers, New York, NY, USA).

### 2.6. The Psychometric Properties of WISC-IV

Inter-subscale correlations were analyzed and compared between the two groups. The overall Cronbach’s alpha of these 84 participants was 0.790, and the domain reliabilities were VCI 0.861, PRI 0.720, WMI 0.686, and PSI 0.691. Supplementary Table S1 indicated the correlation patterns of correlation coefficients among domains and total score. In both groups, the four domains were significantly correlated with the total scores (all *p* < 0.05). However, the PSI–FSIQ correlation coefficient was significantly higher in the exotropia group than in the normal group (0.759 vs. 0.464, *p* = 0.032). In the pattern of domain correlations, there were more significant correlations among the four domains in the exotropia group than in the normal group.

As for the difference between domains, the significant correlation patterns were inconsistent between two groups in the PRI−WMI and VCI−PRI; PRI−WMI and VCI−WMI; PRI−PSI and PRI−WMI; and WMI−PSI and VCI−PSI. These results indicated that the exotropia children had a different intelligence structure and correlation among domains as compared with the normal children.

## 3. Results

### 3.1. Demographic and Baseline Clinical Characteristics

A total of 84 participants were recruited into this study, including 37 patients (mean age: 10.3 ± 1.4; 21 males and 16 females) with exotropia and 47 normal individuals (mean age: 10.13 ± 1; 28 males and 19 females). All the 84 participants completed the questionnaires. The demographic and clinical characteristics were compared between the exotropia group and the normal groups (Table 1).

In the exotropia group, 23 (62.2%) cases had intermittent exotropia (X(T)) and 14 (37.8%) cases had constant exotropia (XT). There were 26 (70.3%) cases with near stereoacuity and 10 (27.0%) cases with distance stereoacuity. The mean strabismus angle was 31.8 ± 10.0 prism diopters (PD), and the duration of symptoms was 4.6 ± 2.6 years.

As shown in Table 1, there were significant differences in the place of residence (borderline statistical difference: *p* = 0.057), education level of mother (*p* < 0.001), education level of father, and total monthly household income between the exotropia group and the normal group (*p* < 0.001). These factors would be adjusted as covariates in multiple linear regression. No significant differences were found in other demographic and clinical characteristics.

### 3.2. Comparison of WISC-IV Score between the Exotropia Group and the Normal Group

The WISC-IV score of both groups is shown in Table 2. Comparison of the WISC-IV score showed that the exotropia group had a significantly lower PRI score (*p* = 0.003) but higher PSI score (*p* = 0.034) than the normal group. However, there was no significant difference in the WMI, VCI, and FSIQ between two groups (all *p* > 0.05). Intragroup comparison among the four domains showed that the exotropia group had a pattern of VCI > PSI > WMI > PRI, while the normal group had a pattern of VCI > PRI > WMI > PSI (Table 2).

### 3.3. Comparison of Subtest-Level Discrepancy between the Exotropia Group and the Normal Group

Subtest-level discrepancy comparison was calculated by the differences between each index, which can provide an advantage pattern of intelligence structure. Linear regression models were used to examine the subtest-level discrepancy between the exotropia group and the normal groups. 

As shown in Table 3, the exotropia group had higher VCI−PRI score after adjusting for household characteristics (*p* < 0.014). The PRI−WMI and PRI−PSI were significantly different in both the simple and multiple linear regression (all *p* < 0.01).

However, significant differences in VCI−WMI and VCI−PSI between the two groups were only observed in the simple linear regression. The WMI−PSI score was not significantly different between the two groups.

The distribution of subtest-level differences was compared between groups. The exotropia group had significantly more cases with a minus difference in the VCI−PSI (*p* = 0.029) and PRI−PSI (*p* = 0.001) differences than the normal group (Table S2), indicating that PSI was higher in the exotropia group.

### 3.4. Subgroup Analysis Stratified by Type of Exotropia

To investigate if the type of exotropia has an effect on intelligence structures, WISC-IV scores were compared between the XT patients (n = 14) and X(T) patients (n = 23)**.** As shown in Table 4, XT patients had significantly higher VCI, WMI, PSI, and FSIQ than X(T) patients (all *p* < 0.05). However, subtest-level discrepancy comparison showed that there was no significant difference in all the six subtest-level discrepancies between the two groups (Table 5, all *p* > 0.05).

### 3.5. Relationship between Intelligence Structures and Angle of Deviation/Duration of Symptoms

Next, we determined the effect of angle of deviation and duration of symptoms on intelligence structures. The results showed that all the six subtest-level discrepancies had no significant linear associations with the angle of deviation (Table 6) and duration of symptoms (Table 7) in both simple and multiple results (all *p* > 0.05).

### 3.6. Relationship between Intelligence Structures and Near Stereoacuity

The effect of near/distance stereoacuity on intelligence structures was investigated. The 37 exotropia patients were sub-grouped into the near stereoacuity (n = 26) subgroup and those without near stereoacuity (n = 11). As shown in Table 4, there was no significant difference in all WISC-IV indexes between the two groups. In addition, no significant difference was found in the six subtest-level discrepancies between the two subgroups in both mean comparisons and simple linear regression results (Table 8, all *p* > 0.05). Since there was no significant difference in demographic variables, further multiple adjustment of covariate was not performed.

Subgroup analysis of distance stereoacuity was also analyzed. As shown in Table 4, patients with distance stereoacuity had a significantly lower PRI (n = 10) than those without distance stereoacuity (n = 27) (*p* = 0.031). However, all the six subtest-level discrepancies had no significant linear associations with distance stereoacuity in both simple and multiple results (Table 9, all *p* > 0.05).

## 4. Discussion

Intelligence is of significant value to children’s adaptation to the environment and future development. Therefore, understanding the intelligence development of exotropia children is helpful to better understand the adverse effect of exotropia, promptly conduct the intervention on cognitive deficits, and prevent learning disabilities and secondary problems. In addition, during intervention of exotropia, the improvement of intelligence level might be one of the prognostic indicators.

In this study, we characterized intelligence in children with exotropia by WISC-IV. The results showed that the exotropia group had a significantly lower PRI score but a higher PSI score than the normal group. However, there was no significant difference in the WMI, VCI, and FSIQ between the two groups. Multiple linear regression showed that PRI−WMI and PRI−PSI differences were significantly lower in the exotropia group than in the normal group. Inter-subscale correlation analysis also confirmed that the exotropia children had different intelligence structures as compared with normal children. The type of exotropia, angle of deviation, duration of symptoms, and stereoacuity had no effect on the intelligence of children with exotropia. Taken together, these results suggest that children with exotropia had a relatively worse performance in the perceptual reasoning skill but a better processing speed and a different pattern of intelligence structure as compared with normal children.

Our results demonstrated that the exotropia group had a significantly lower PRI score than the normal group. The core subtests of PRI include Block Design, Picture Concept, and Matrix Reasoning, which are mainly used to assess the abilities of visual–spatial information processing, visual action integration, and perceptual fluid reasoning. Accumulating evidence has suggested that strabismus has a significant impact on visual perception and visuomotor behavior [26,27,28]. Meanwhile, exotropia causes several adverse effects on visual function, including visual suppression, abnormal retinal correspondence, and diplopia [29,30]. Bertone et al. have found that healthy adult participants with lower visual acuity have worse performance in perceptual reasoning and visual search tests, suggesting perceptual reasoning skills are affected by visual degradation [31]. Therefore, the exotropia-induced adverse effects on visual function may contribute to the declined perceptual reasoning ability. We found that 29.73% and 72.97% exotropia children lost their near stereoacuity and distance stereoacuity, respectively, suggesting that exotropia was more harmful to distance stereoacuity than near stereoacuity. This observation is in line with a previous study [32]. Since stereoscopic vision provides an important source of depth perception [33], the reduced stereoscopic vision in exotropia children may also contribute to the decrease in perceptual reasoning ability. Exotropia also leads to interocular suppression, which leads to perceptual distortion and affects the perceptual integration in the primary visual cortex (V1), in turn affecting the perceptual reasoning ability [34].

PSI is the assessment of children’s ability to process simple visual information quickly, including the subtests of Coding and Symbol Search. Although Bertone et al. report that processing speed ability is affected by visual degradation in healthy adults [31], our results revealed that exotropia children had a higher PSI than normal children. An eye movement study shows that strabismus patients have comparable accuracy and precision of visually guided reaching movements and the total movement time with normal individuals [27]. Because the total field of vision is expanded by ocular deviation, exotropic individuals experience a more panoramic view[28,35], which might bring some advantages to children with exotropia when dealing with simple visual information. On the other hand, exotropia-induced interocular suppression can lead to unilateral fixation, and thereby the transmission of visual information and the processing speed are improved [34]. However, interocular suppression also affects the perceptual integration ability. Therefore, we observed an improvement in processing speed but a decline in the perceptual reasoning ability in exotropia children. Nevertheless, the mechanism underlying the improvement of processing speed by exotropia remains to be further elucidated. Moreover, due to the small sample size of the current study, this finding should be validated in a larger sample size. Since the exotropia children had weakness in PRI and strength in PSI, subtest-level discrepancy comparisons showed that the exotropia group had a significantly lower PRI−WMI and PRI−PSI differences than the normal group, suggesting that exotropia children presented a different intelligence structure. Comparison of the distribution of subtest-level differences showed that the exotropia group had more cases with minus difference in the VCI−PSI and PRI−PSI than the normal group, indicating that PSI was higher in the exotropia group. Moreover, our inter-subscale correlations analysis identified a different pattern of significant correlations between the exotropia and normal groups, further supporting that the exotropia children had different intelligence structures as compared with normal children. 

In this study, XT patients had higher IQ scores than X(T) patients, which is consistent with previous studies [17,18]. It has been shown that most XT patients are deteriorated from X(T) [36], and XT may present decompensated X(T) [37], indicating that XT and X(T) have the same etiological basis. This may explain our observation that there was no significant difference in the pattern of intelligence structure between XT and X(T) in the subtest−level discrepancy comparison. Our analysis showed that the angle of deviation had no effect on intelligence in exotropia children. This finding is in line with the previous observation that there is no correlation between deviation severity and psychological parameters in exotropia patients [38]. During the pathogenesis of exotropia, the stereoacuity, control, and angle of deviation are unstable, and exotropia children may exhibit any combination of stereoacuity, control, and angle of deviation [39]. The instability of the squint angle may lead to no correlation between intelligence and the angle of deviation. As for stereoscopic vision, Gligorovic et al. have demonstrated that visually impaired children without stereoscopic vision have poor non-verbal reasoning abilities [21]. However, we did not observe this phenomenon. Our subgroup analysis showed that there was no significant difference in the WISC-IV score between the exotropia patients with or without near stereoacuity. Although children without distance stereoacuity showed a higher PRI than those with distance stereoacuity, the subtest-level discrepancy comparisons did not show any difference between groups. This discrepancy may be attributed to our small sample size. 

There are still some limitations of this study. First, the sample size of this study was relatively small. In addition, all of the participants in the exotropia group were recruited from patients scheduled for surgery, and these patients had a more severe disease condition, such as longer duration of symptoms, larger squint angles, and poor stereopsis. Hence, the selection bias may make the findings of this study unable to be applicable to all exotropia children. Furthermore, we did not evaluate postoperative IQ to determine the effect of surgery on intelligence. Previous studies have reported beneficial effects of strabismus surgery on quality of life and mental health [40,41]. Treating strabismus timely not only protects stereoscopic vision but also enhances the social interaction ability of patients [42,43]. In the future, a well-designed prospective clinical trial with a large sample size should be conducted to validate the findings of this study and address these limitations.

## 5. Conclusions

In summary, the current study suggested that compared to normal children, exotropia children had a relatively worse performance in the perceptual reasoning skill but a better processing speed and a different pattern of intelligence structure. Type of exotropia, the angle of deviation, duration of symptoms, and stereoacuity did not affect the intelligence of children with exotropia.

## Figures and Tables

**Table 1:** Demographic and baseline clinical characteristics.

Variables	Total (n = 84)	Normal (n = 47)	Exotropia (n = 37)	*p* ^a^
Age (in years)	10.19 ± 1.26	10.13 ± 1.14	10.26 ± 1.40	0.656
Gender				
Male	49 (58.33)	28 (59.57)	21 (56.76)	0.795
Female	35 (41.67)	19 (40.43)	16 (43.24)	
Place of residence				
Country	23 (27.38)	9 (19.15)	14 (37.84)	0.057
City	61 (72.62)	38 (80.85)	23 (62.16)	
Education level of mother				
Below high school	10 (11.90)	2 (4.26)	8 (21.62)	<0.001
High school or equivalent	22 (26.19)	7 (14.89)	15 (40.54)	
College undergraduate	43 (51.19)	29 (61.70)	14 (37.84)	
Bachelor degree or above	9 (10.71)	9 (19.15)	0 (0.00)	
Education level of father				
Below high school	7 (8.33)	1 (2.13)	6 (16.22)	<0.001
High school or equivalent	19 (22.62)	5 (10.64)	14 (37.84)	
College undergraduate	42 (50.00)	29 (61.70)	13 (35.14)	
Bachelor degree or above	16 (19.05)	12 (25.53)	4 (10.81)	
Total monthly household income				
Less than 10,000 RMB	19 (22.62)	4 (8.51)	15 (40.54)	<0.001
10,000–19,999 RMB	26 (30.95)	13 (27.66)	13 (35.14)	
20,000–29,999 RMB	19 (22.62)	17 (36.17)	2 (5.41)	
More than 30,000 RMB	20 (23.81)	13 (27.66)	7 (18.92)	
With or without sibling(s)				
Without	41 (48.81)	27 (57.45)	14 (37.84)	0.074
With	43 (51.19)	20 (42.55)	23 (62.16)	
Gestational age				
Full-term	76 (90.48)	41 (87.23)	35 (94.59)	0.254
Pre-term	8 (9.52)	6 (12.77)	2 (5.41)	
Delivery method				
Natural childbirth	42 (50.00)	21 (44.68)	21 (56.76)	0.272
Cesarean section	42 (50.00)	26 (55.32)	16 (43.24)	
Admitted to NICU at birth				
None	78 (92.86)	45 (95.74)	33 (89.19)	0.247
Once	6 (7.14)	2 (4.26)	4 (10.81)	
Best vision				0.047
≤0.8	3 (3.57)	3 (8.11)	0 (0.00)	
>0.8	81 (96.43)	34 (91.89)	47 (100.00)	
Disease category				-
Normal	-	47 (100.00)	-	
Intermittent exotropia	-	-	23 (62.16)	
Constant exotropia	-	-	14 (37.84)	
Near stereoacuity				-
Have	-	-	26 (70.27)	
None	-	-	11 (29.73)	
Distance stereoacuity				-
Have	-	-	10 (27.03)	
None	-	-	27 (72.97)	
Angle of deviation	-	-	31.82 ± 10.03	-
Duration of symptoms (in years)	-	-	4.59 ± 2.65	-

^a^ Chi-square Test of equal proportions for categorical variables; t test for continuous variables.

**Table 2:** Comparison of WISC-IV scores between the exotropia group and the normal group.

WISC-IV Score	Total (n = 84)	Normal (n = 47)	Exotropia (n = 37)	*p* ^a^
VCI	115.08 ± 17.05	118.66 ± 15.81	110.54 ± 17.68	0.889
PRI	106.13 ± 13.71	110.32 ± 13.06	100.81 ± 12.80	0.003
WMI	103.17 ± 14.71	102.96 ± 14.75	103.43 ± 14.86	0.231
PSI	103.45 ± 13.58	101.26 ± 9.75	106.24 ± 17.00	0.034
FSIQ	109.70 ± 13.65	111.77 ± 11.76	107.08 ± 15.50	0.990

WISC-IV, Wechsler Intelligence Scale for Children, the fourth edition; VCI, Verbal Comprehension Index; PRI, Perceptual Reasoning Index; WMI, Working Memory Index; PSI, Processing Speed Index; FSIQ, Full Scale Intelligence Quotient. ^a^ Adjusted for place of residence, education level of mother, education level of fat her, total monthly household income.

**Table 3:** Comparison of subtest-level discrepancy between the exotropia group and the normal group.

Variables	Mean ± SD		Simple Linear Regression *^a^*			Multiple Linear Regression ^a^		
	Normal (n = 47)	Exotropia (n = 37)	*B*	95% CI	*p*	*B*	95% CI	*p ^b^*
VCI−PRI	8.34 ± 18.68	9.73 ± 18.94	1.39	(−6.83, 9.61)	0.738	10.68	(2.22, 19.13)	0.014
VCI−WMI	15.70 ± 20.76	7.11 ± 18.19	−8.59	(−17.19, 0.01)	0.050	−3.74	(−13.00, 5.53)	0.425
VCI−PSI	17.40 ± 17.90	4.30 ± 19.21	−13.11	(−21.19, −5.02)	0.002	−6.74	(−15.57, 2.09)	0.133
PRI−WMI	7.36 ± 15.26	−2.62 ± 12.52	−9.98	(−16.16, −3.81)	0.002	−14.41	(−21.41, −9.13)	<0.001
PRI−PSI	9.06 ± 14.54	−5.43 ± 15.43	−14.50	(−21.03, −7.97)	<0.001	−17.42	(−25.05, −9.79)	<0.001
WMI−PSI	1.70 ± 15.79	−2.81 ± 16.57	−4.51	(−11.57, 2.54)	0.207	−3.00	(−11.22, 5.21)	0.469

VCI, Verbal Comprehension Index; PRI, Perceptual Reasoning Index; WMI, Working Memory Index; PSI, Processing Speed Index. ^a^ Normal group as the reference; ^b^ adjusted for place of residence, education level of mother, education level of father, and total monthly household income.

**Table 4:** Subgroup analysis of the WISC-IV index scores stratified by different types of exotropia, near stereoacuity, and distance stereo acuity.

Variables		VCI	PRI	WMI	PSI	FSIQ
Type of exotropia						
X(T) (n = 23)	Mean ± SD	108.13 ± 15.35	98.43 ± 14.26	101.26 ± 14.34	102.39 ± 13.90	103.61 ± 14.50
XT (n = 14)		114.50 ± 20.98	104.71 ± 9.14	107.00 ± 15.52	112.57 ± 20.11	112.79 ± 15.91
	*t*	−1.06	−1.47	−1.14	−1.82	−1.80
	*p*	0.294	0.150	0.260	0.077	0.081
	*p ^a^*	0.012	0.060	0.048	0.037	0.003
Near stereoacuity						
Have (n = 26)	Mean ± SD	107.58 ± 15.59	99.50 ± 14.10	101.58 ± 14.40	105.31 ± 16.28	104.85 ± 14.83
None (n = 11)		117.55 ± 21.02	103.91 ± 8.84	107.82 ± 15.68	108.45 ± 19.26	112.36 ± 16.49
	*t*	−1.60	−0.96	−1.17	−0.51	−1.36
	*p*	0.118	0.345	0.248	0.614	0.181
Distance stereoacuity						
Have (n = 10)	Mean ± SD	111.20 ± 22.18	95.30 ± 10.51	103.10 ± 14.16	99.60 ± 14.32	102.90 ± 17.01
None (n = 27)		110.30 ± 16.20	102.85 ± 13.14	103.56 ± 15.37	108.70 ± 17.50	108.63 ± 14.95
	*t*	0.14	−1.63	−0.08	−1.47	−1.00
	*p*	0.892	0.112	0.935	0.151	0.325
	*p ^b^*	0.979	0.031	0.504	0.080	0.130

VCI, Verbal Comprehension Index; PRI, Perceptual Reasoning Index; WMI, Working Memory Index; PSI, Processing Speed Index; FSIQ, Full Scale Intelligence Quotient. ^a^ Adjusted for place of residence, education level of father, and gestational age. ^b^ Adjusted for age and education level of father.

**Table 5:** Comparison of subtest-level discrepancy between the XT and X(T) patients.

Variables	Mean ± SD		Simple Linear Regression ^a^			Multiple Linear Regression ^a^		
	X(T) (n = 23)	XT (n = 14)	*B*	95% CI	*p*	*B*	95% CI	*p ^b^*
VCI−PRI	9.70 ± 19.12	9.79 ± 19.35	0.09	(−13.13, 13.31)	0.989	6.41	(−7.78, 20.60)	0.365
VCI−WMI	6.87 ± 14.82	7.50 ± 23.33	0.63	(−12.06, 13.32)	0.920	5.46	(−7.84, 18.77)	0.409
VCI−PSI	5.74 ± 18.21	1.93 ± 21.24	−3.81	(−17.15, 9.53)	0.566	2.07	(−12.39, 16.53)	0.773
PRI−WMI	−2.83 ± 11.77	−2.29 ± 14.12	0.54	(−8.20, 9.28)	0.901	−0.94	(−10.27, 20.60)	0.838
PRI−PSI	−3.96 ± 11.21	−7.86 ± 20.88	−3.90	(−14.58, 6.78)	0.464	−4.34	(−16.53, 7.85)	0.474
WMI−PSI	−1.13 ± 14.07	−5.57 ± 20.30	−4.44	(−15.90, 7.02)	0.437	−3.40	(−16.46, 9.66)	0.600

XT, constant exotropia; X(T), intermittent exotropia; VCI, Verbal Comprehension Index; PRI, Perceptual Reasoning Index; WMI, Working Memory Index; PSI, Processing Speed Index. ^a^ Select intermittent exotropia group as control group; ^b^ adjusted for place of residence, education level of father, and gestational age.

**Table 6:** The regression analysis between subtest-level discrepancy and angle of deviation in the exotropia group.

Variables	Simple Linear Regression			Multiple Linear Regression ^a^		
	*B*	95% CI	*p*	*B*	95% CI	*p*
VCI−PRI	−0.09	(−0.73, 0.56)	0.790	−0.05	(−0.66, 0.56)	0.870
VCI−WMI	−0.22	(−0.84, 0.39)	0.466	−0.22	(−0.81, 0.38)	0.463
VCI−PSI	−0.38	(−1.02, 0.26)	0.239	−0.25	(−0.87, 0.37)	0.416
PRI−WMI	−0.14	(−0.56, 0.29)	0.513	−0.17	(−0.67, 0.33)	0.499
PRI−PSI	−0.29	(−0.81, 0.22)	0.256	−0.20	(−0.85, 0.45)	0.536
WMI−PSI	−0.16	(−0.72, 0.41)	0.578	−0.03	(−0.76, 0.70)	0.927

VCI, Verbal Comprehension Index; PRI, Perceptual Reasoning Index; WMI, Working Memory Index; PSI, Processing Speed Index. ^a^ Adjusted for gender, age, place of residence, education level of mother, education level of father, total monthly household income, gestational age, with or without sibling(s), and delivery method.

**Table 7:** The regression analysis between subtest-level discrepancy and duration of symptoms in the exotropia group.

Variables	Simple Linear Regression			Multiple Linear Regression ^a^		
	*B*	95% CI	*p*	*B*	95% CI	*p*
VCI−PRI	−0.89	(−3.33, 1.54)	0.461	−0.14	(−2.30, 2.03)	0.899
VCI−WMI	−0.87	(−3.21, 1.47)	0.455	0.01	(−2.13, 2.15)	0.995
VCI−PSI	−1.19	(−3.64, 1.27)	0.333	−0.44	(−2.65, 1.78)	0.689
PRI−WMI	0.02	(−1.60, 1.64)	0.978	0.14	(−1.65, 1.94)	0.871
PRI−PSI	−0.29	(−2.29, 1.70)	0.767	−0.30	(−2.64, 2.04)	0.793
WMI−PSI	−0.32	(−2.46, 1.83)	0.766	−0.44	(−3.03, 2.14)	0.727

VCI, Verbal Comprehension Index; PRI, Perceptual Reasoning Index; WMI, Working Memory Index; PSI, Processing Speed Index. ^a^ Adjusted for gender, age, pace of residence, education level of mother, education level of father, total monthly household income, gestational age, with or without sibling(s), and delivery method.

**Table 8:** The regression analysis between subtest-level discrepancy and near stereoacuity in the exotropia group.

Variables	Mean ± SD		Simple Linear Regression ^a^		
	Have (n = 26)	None (n = 11)	*B*	95%CI	*p*
VCI−PRI	8.08 ± 18.84	13.64 ± 19.49	5.56	(−8.33, 19.45)	0.422
VCI−WMI	6.00 ± 17.30	9.73 ± 20.79	3.73	(−9.68, 17.14)	0.576
VCI−PSI	2.27 ± 19.88	9.09 ± 17.46	6.82	(−7.21, 20.85)	0.330
PRI−WMI	−2.08 ± 11.80	−3.91 ± 14.63	−1.83	(−11.08, 7.42)	0.690
PRI−PSI	−5.81 ± 12.79	−4.55 ± 21.13	1.26	(−10.15, 12.68)	0.824
WMI−PSI	−3.73 ± 15.67	−0.64 ± 19.15	3.09	(−9.13, 15.32)	0.611

VCI, Verbal Comprehension Index; PRI, Perceptual Reasoning Index; WMI, Working Memory Index; PSI, Processing Speed Index. ^a^ With near stereo acuity group as the reference; the distribution of demographic information is consistent in the two groups, and multiple regression analysis is not needed.

**Table 9:** The regression analysis between subtest-level discrepancy and distance stereoacuity in the exotropia group.

Variables	Mean ± SD		Simple Linear Regression ^a^			Multiple Linear Regression ^a^		
	Have (n = 10)	None (n = 27)	*B*	95%CI	*p*	*B*	95%CI	*p^b^*
VCI−PRI	15.90 ± 18.63	7.44 ± 18.87	−8.46	(−22.59, 5.68)	0.233	−11.10	(−26.07, 3.87)	0.141
VCI−WMI	8.10 ± 17.93	6.74 ± 18.61	−1.36	(−15.22, 12.50)	0.843	−3.96	(−19.04, 11.12)	0.597
VCI−PSI	11.60 ± 21.18	1.59 ± 18.10	−10.01	(−24.24, 4.22)	0.162	−12.09	(−27.60, 3.42)	0.122
PRI−WMI	−7.80 ± 12.81	−0.70 ± 12.09	7.10	(−2.13, 16.32)	0.128	7.14	(−2.31, 16.59)	0.134
PRI−PSI	−4.30 ± 10.86	−5.85 ± 16.97	−1.55	(−13.30, 10.19)	0.790	−0.99	(−13.78, 11.80)	0.876
WMI−PSI	3.50 ± 11.01	−5.15 ± 17.81	−8.65	(−20.92, 3.63)	0.162	−8.13	(−21.56, 5.30)	0.227

VCI, Verbal Comprehension Index; PRI, Perceptual Reasoning Index; WMI, Working Memory Index; PSI, Processing Speed Index. ^a^ With distance stereo acuity group as the reference; ^b^ adjusted for age and education level of father.

## Supplementary Materials

The following are available online at https://www.mdpi.com/1660-4601/16/17/3008/s1, Table S1: Comparison of the distribution of subtest-level differences between groups, Table S2: The correlation analysis results among domains and total score (FSIQ) or among differences.

## References

[1] Green-Simms A.E., Mohney B.G. (2010). Epidemiology of Pediatric Strabismus.

[2] Bruce A., Santorelli G. (2016). Prevalence and Risk Factors of Strabismus in a UK Multi-ethnic Birth Cohort. Strabismus.

[3] Hashemi H., Pakzad R., Heydarian S., Yekta A., Aghamirsalim M., Shokrollahzadeh F., Khoshhal F., Pakbin M., Ramin S., Khabazkhoob M. (2019). Global and regional prevalence of strabismus: A comprehensive systematic review and meta−analysis. Strabismus.

[4] Hashemi H., Yekta A., Jafarzadehpur E., Ostadimoghaddam H., Eshrati B., Mohazzab-Torabi S., Khabazkhoob M., Soroush S. (2015). The prevalence of strabismus in 7-year-old schoolchildren in Iran. Strabismus.

[5] McKean-Cowdin R., Cotter S.A., Tarczy-Hornoch K., Wen G., Kim J., Borchert M., Varma R., Multi-Ethnic Pediatric Eye Disease Study Group (2013). Prevalence of amblyopia or strabismus in asian and non-Hispanic white preschool children: Multi-ethnic pediatric eye disease study. Ophthalmology.

[6] Torp-Pedersen T., Boyd H.A., Skotte L., Haargaard B., Wohlfahrt J., Holmes J.M., Melbye M. (2017). Strabismus Incidence in a Danish Population-Based Cohort of Children. JAMA Ophthalmol..

[7] Yu X., Ji Z., Yu H., Xu M., Xu J. (2016). Exotropia Is the Main Pattern of Childhood Strabismus Surgery in the South of China: A Six-Year Clinical Review. J. Ophthalmol..

[8] Zhu H., Pan C., Sun Q., Huang D., Fu Z., Wang J., Chen X., Wang Z., Liu H. (2019). Prevalence of amblyopia and strabismus in Hani school children in rural southwest China: A cross-sectional study. BMJ Open.

[9] Pi L.H., Chen L., Liu Q., Ke N., Fang J., Zhang S., Xiao J., Ye W.J., Xiong Y., Shi H. (2012). Prevalence of Eye Diseases and Causes of Visual Impairment in School-Aged Children in Western China. J. Epidemiol..

[10] Chen X., Fu Z., Yu J., Ding H., Bai J., Chen J., Gong Y., Zhu H., Yu R., Liu H. (2016). Prevalence of amblyopia and strabismus in Eastern China: Results from screening of preschool children aged 36−72 months. Br. J. Ophthalmol..

[11] Cui J., Zhang Y., Wan S., Chen C., Zeng J., Zhou X. (2019). Visual form perception is fundamental for both reading comprehension and arithmetic computation. Cognition.

[12] Lipton E.L. (1970). A study of the psychological effects of strabismus. Psychoanal. Study Child.

[13] Tonge B.J., Lipton G.L., Crawford G. (1984). Psychological and educational correlates of strabismus in school children. Aust. N. Z. J. Psychiatry..

[14] Granet D.B., Gomi C.F., Ventura R., Miller-Scholte A. (2005). The relationship between convergence insufficiency and ADHD. Strabismus.

[15] Chung S.A., Chang Y.H., Rhiu S., Lew H., Lee J.B. (2012). Parent-reported symptoms of attention deficit hyperactivity disorder in children with intermittent exotropia before and after strabismus surgery. Yonsei. Med. J..

[16] Merdler I., Giladi M., Sorkin N., Shapira S., Galili E., Margulis A., Korach T., Hassidim A. (2017). Strabismus and mental disorders among Israeli adolescents. J. AAPOS.

[17] Bagheri A., Fallahi M.R., Tamannaifard S., Vajebmonfared S., Zonozian S. (2013). Intelligence Quotient (IQ) in Congenital Strabismus. J. Ophthalmic. Vis. Res..

[18] Ghaderpanah M., Farrahi F., Khataminia G., Jahanbakhshi A., Rezaei L., Tashakori A., Mahboubi M. (2015). Comparing Intelligence Quotient (IQ)among 3 to 7-year-old strabismic and nonstrabismic children in an Iranian population. Glob. J. Health Sci..

[19] Read J.C. (2015). A Stereo vision and strabismus. Eye.

[20] Zhu P.W., Huang X., Ye L., Jiang N., Zhong Y.L., Yuan Q., Zhou F.Q., Shao Y. (2018). Altered intrinsic functional connectivity of the primary visual cortex in youth patients with comitant exotropia: A resting state fMRI study. Int. J. Ophthalmol..

[21] Gligorović M., Vučinić V., Eškirović B., Jablan B. (2011). The influence of manifest strabismus and stereoscopic vision on non-verbal abilities of visually impaired children. Res. Dev. Disabil..

[22] Flanagan D.P., Kaufman A.S. (2009). Essentials of WISC-IV Assessment.

[23] (2015). The strabismus and pediatric ophthalmology group OB of CMA Chinese expert consensus on strabismus classification. Chin. J. Ophthalmol..

[24] Zhang H. (2009). The revision of WISC-IV Chinese version. Psychol. Sci..

[25] Wang J., Zou Y.Z., Cui J.F., Fan H.Z., Chen N., Yao J., Duan J.H., Chen R., Yan L.J., He X.L. (2013). Reliability and construct validity of the Chinese version of the Wechsler Adult Intelligence Scale−Fourth Edition. Chinese Ment. Heal. J..

[26] Fazzi E., Bova S.M., Uggetti C., Signorini S.G., Bianchi P.E., Maraucci I., Zoppello M., Lanzi G. (2004). Visual-perceptual impairment in children with periventricular leukomalacia. Brain Dev..

[27] Dadeya S., Dangda S., Piano M.E., Tidbury L.P., O’Connor A.R., Duffy K.R., Bukhamseen D.H., Smithen M.J., Mitchell D.E., Hu M. (2014). Effects of strabismic amblyopia on visuomotor behavior: Part II. Visually guided reaching. Vision Res..

[28] Sayyadi S., Lajevardi L., Aliabadi F., Keihani M.R., Abbasi L. (2011). Comparing visual perceptual skills among 8 to 10-year-old strabismal/nonstrabismal cerebral palsy children. Feyz. J. Kashan. Univ. Med. Sci..

[29] Scholl B., Tan A.Y., Priebe N.J. (2013). Strabismus Disrupts Binocular Synaptic Integration in Primary Visual Cortex. J. Neurosci..

[30] Economides J.R., Adams D.L., Horton J.C. (2012). Perception via the deviated eye in strabismus. J. Neurosci..

[31] Bertone A., Bettinelli L., Faubert J. (2007). The impact of blurred vision on cognitive assessment. J. Clin. Exp. Neuropsychol..

[32] Li S., Zhao R., Huang X., Wei C. (2010). Clinical study of binocular vision in intermittent and constant exotropia. Recent Adv. Ophthalmol..

[33] Hibbard P.B., Haines A.E., Hornsey R.L. (2017). Magnitude, precision, and realism of depth perception in stereoscopic vision. Cogn. Res. Princ. Implic..

[34] Sengpiel F., Blakemore C. (1996). The neural basis of suppression and amblyopia in strabismus. Eye.

[35] Serrano-Pedraza I., Manjunath V., Osunkunle O., Clarke M.P., Read J.C. (2011). Visual suppression in intermittent exotropia during binocular alignment. Investig. Ophthalmol. Vis. Sci..

[36] Livir-Rallatos G., Gunton K.B., Calhoun J.H. (2002). Surgical results in large-angle exotropia. J. AAPOS.

[37] Abroms A.D., Mohney B.G., Rush D.P., Parks M.M., Tong P.Y. (2001). Timely surgery in intermittent and constant exotropia for superior sensory outcome. Am. J. Ophthalmol..

[38] Lim S.B., Wong W.L., Ho R.C., Wong I.B. (2015). Childhood intermittent exotropia from a different angle: Does severity affect quality of life?. Br. J. Ophthalmol..

[39] Superstein R., Dean T.W., Holmes J.M., Chandler D.L., Cotter S.A., Wallace D.K., Melia B.M., Kraker R.T., Weaver R.G., Mohney B.G. (2017). Relationship among clinical factors in childhood intermittent exotropia. J. AAPOS.

[40] Alpak G., Coskun E., Erbagci I., Bez Y., Okumus S., Oren B., Gurler B. (2014). Effects of corrective surgery on social phobia, psychological distress, disease−related disability and quality of life in adult strabismus patients. Br. J. Ophthalmol..

[41] Gunton K.B. (2014). Impact of strabismus surgery on health-related quality of life in adults. Curr. Opin. Ophthalmol..

[42] Kattan J.M., Velez F.G., Demer J.L., Pineles S.L. (2016). Relationship between Binocular Summation and Stereoacuity after Strabismus Surgery. Am. J. Ophthalmol..

[43] Pineles S.L., Demer J.L., Isenberg S.J., Birch E.E., Velez F.G. (2015). Improvement in binocular summation after strabismus surgery. JAMA Ophthalmol..

