# Supplementary material for: Characterization of Intelligence in Children with Exotropia
Source: Int J Environ Res Public Health. 2019 Aug 21;16(17):3008. doi: 10.3390/ijerph16173008 (PMC6747520; doi:10.3390/ijerph16173008)
Supplement: Supplementary file 1 [file ijerph-16-03008-s001.pdf]

Supplementary Table S1. Comparison of the distribution of subtest-level differences between groups

		No difference	Positive difference	Minus difference
VCI-PRI	Normal	24 (51.1)	18 (38.3)	5 (10.6)
N (%)	Exotropia	13 (35.1)	18 (48.6)	6 (16.2)
	X^2	2.218		
	P	0.330		
VCI-WMI	Normal	11 (23.4)	31 (66.0)	5 (10.6)
N (%)	Exotropia	15 (40.5)	16 (43.2)	6 (16.2)
	X^2	4.388		
	P	0.111		
VCI-PSI	Normal	19 (40.4)	26 (55.3)	2 (4.3)
N (%)	Exotropia	18 (48.6)	12 (32.4)	7 (18.9)
	X^2	7.057		
	P	0.029		
PRI-WMI	Normal	22 (46.8)	18 (38.3)	7 (14.9)
N (%)	Exotropia	21 (56.8)	6 (16.2)	10 (27.0)
	X^2	5.439		
	P	0.066		
PRI-PSI	Normal	27 (57.4)	18 (38.3)	2 (4.3)
N (%)	Exotropia	22 (59.5)	4 (10.8)	11 (29.7)
	X^2	14.667		
	P	0.001		
WMI-PSI	Normal	32 (68.1)	8 (17.0)	7 (14.9)
N (%)	Exotropia	21 (56.8)	5 (13.5)	11 (29.7)
	X^2	2.712		
	P	0.258		

VCI, Verbal Comprehension Index; PRI; Perceptual Reasoning Index; WMI, Working Memory Index; PSI, Processing Speed Index.

Supplementary Table S2. The correlation analysis results among domains and total score (FSIQ) or among differences

	Correlation coefficients					
WISC-IV domains	VCI	PRI	WMI	PSI	FSIQ	
Normal group						
VCI	-	0.173	0.079	0.080	0.645	
PRI		-	0.403	0.212	0.727	
WMI			-	0.221	0.628	
PSI				-	0.464	
Exotropia group	VCI	PRI	WMI	PSI	FSIQ	
VCI	-	0.261	0.386	0.387	0.737	
PRI		-	0.599	0.494	0.739	
WMI			-	0.466	0.776	
PSI				-	0.759[a]	
Difference between domains						
Normal group	VCI-PRI	VCI-WMI	VCI-PSI	PRI-WMI	PRI-PSI	WMI-PSI
VCI-PRI	-	0.705	0.685	-0.265	-0.442	-0.151
VCI-WMI		-	0.676	0.497	-0.074	-0.549
VCI-PSI			-	0.081	0.351	0.246
PRI-WMI				-	0.440	-0.562
PRI-PSI					-	0.496
Exotropia group	VCI-PRI	VCI-WMI	VCI-PSI	PRI-WMI	PRI-PSI	WMI-PSI
VCI-PRI	-	0.773	0.673	-0.389	-0.390	-0.069
VCI-WMI		-	0.609	0.283	-0.191	-0.392
VCI-PSI			-	-0.134	0.419	0.491
PRI-WMI				-	0.311	-0.466
PRI-PSI					-	0.696

WISC-IV, Wechsler Intelligence Scale for Children, the fourth edition; VCI, Verbal Comprehension Index; PRI; Perceptual Reasoning Index; WMI, Working Memory Index; PSI, Processing Speed Index.

Significant correlation coefficients were indicated with bold font.

[a] $P<0.05$, compared with the corresponding correlation coefficient in the normal group.
